# Impact of severe valvular heart disease in adult congenital heart disease patients

**DOI:** 10.3389/fcvm.2022.983308

**Published:** 2022-11-29

**Authors:** Francesca Graziani, Giulia Iannaccone, Maria Chiara Meucci, Rosa Lillo, Angelica Bibiana Delogu, Maria Grandinetti, Gianluigi Perri, Lorenzo Galletti, Antonio Amodeo, Gianfranco Butera, Aurelio Secinaro, Antonella Lombardo, Gaetano Antonio Lanza, Francesco Burzotta, Filippo Crea, Massimo Massetti

**Affiliations:** ^1^Department of Cardiovascular Medicine, Fondazione Policlinico Universitario Agostino Gemelli IRCCS, Rome, Italy; ^2^Department of Cardiovascular and Pulmonary Sciences, Università Cattolica del Sacro Cuore, Rome, Italy; ^3^Unit of Pediatrics, Pediatric Cardiology, Department of Women and Child Health and Public Health, Fondazione Policlinico Universitario Agostino Gemelli IRCCS, Rome, Italy; ^4^Pediatric Cardiac Surgery Unit, Bambino Gesù Children’s Hospital, IRCCS, Rome, Italy; ^5^Pediatric Cardiology Unit, Bambino Gesù Children’s Hospital, IRCCS, Rome, Italy; ^6^Advanced Cardiovascular Imaging Unit, Department of Imaging, Bambino Gesù Children’s Hospital, IRCCS, Rome, Italy

**Keywords:** valvular heart disease, adult congenital heart disease (ACHD), prognosis, hospitalization, mortality

## Abstract

**Background:**

The clinical impact of valvular heart disease (VHD) in adult congenital heart disease (ACHD) patients is unascertained. Aim of our study was to assess the prevalence and clinical impact of severe VHD (S-VHD) in a real-world contemporary cohort of ACHD patients.

**Materials and methods:**

Consecutive patients followed-up at our ACHD Outpatient Clinic from September 2014 to February 2021 were enrolled. Clinical characteristics and echocardiographic data were prospectively entered into a digitalized medical records database. VHD at the first evaluation was assessed and graded according to VHD guidelines. Clinical data at follow-up were collected. The study endpoint was the occurrence of cardiac mortality and/or unplanned cardiac hospitalization during follow-up.

**Results:**

A total of 390 patients (median age 34 years, 49% males) were included and S-VHD was present in 101 (25.9%) patients. Over a median follow-up time of 26 months (IQR: 12–48), the study composite endpoint occurred in 76 patients (19.5%). The cumulative endpoint-free survival was significantly lower in patients with S-VHD *vs.* patients with non-severe VHD (Log rank *p* < 0.001). At multivariable analysis, age and atrial fibrillation at first visit (*p* = 0.029 and *p* = 0.006 respectively), lower %Sat O_2_, higher NYHA class (*p* = 0.005 for both), lower LVEF (*p* = 0.008), and S-VHD (*p* = 0.015) were independently associated to the study endpoint. The likelihood ratio test demonstrated that S-VHD added significant prognostic value (*p* = 0.017) to a multivariate model including age, severe CHD, atrial fibrillation, %Sat O2, NYHA, LVEF, and right ventricle systolic pressure > 45 mmHg.

**Conclusion:**

In ACHD patients, the presence of S-VHD is independently associated with the occurrence of cardiovascular mortality and hospitalization. The prognostic value of S-VHD is incremental above other established prognostic markers.

## Background

The improvement of neonatal and pediatric cardiac care have led to the progressive increase of children with congenital heart disease (CHD) surviving to late adulthood (1), with a significant increase in the healthcare burden worldwide (2). Most adults with CHD (ACHD) retain a lifelong risk of cardiovascular complications, which is related both to the original defects and the possible sequelae of the cardiac surgery performed in childhood. Consequently, the risk of hospitalizations and mortality in ACHD patients remains higher than that of the general population (3, 4). Arrhythmias, heart failure (HF) and need for interventions on valvular heart diseases (VHD) are often part of the ACHD clinical history (5). Notably, in the large spectrum of ACHD, VHD are frequently encountered as primitive congenital lesions, post-surgery sequelae or as acquired new lesions (5).

Although the presence of VHD in ACHD represents a clinical challenge, most data on their relevance come from registries (6) and surveys (7) where the characterization of VHD was limited and their prognostic impact was not ascertained.

In the present study, we assessed the prevalence and clinical impact of severe VHD (S-VHD) in a real-world contemporary cohort of ACHD patients.

## Materials and methods

### Study design and population

This is a single center observational clinical study reporting data that have been prospectively collected within the framework of clinical pathway dedicated to ACHD patients at Fondazione Policlinico Universitario “A. Gemelli,” which is a surgical/interventional tertiary Center and represents a national referral for ACHD patients and for heart valve disease.

For the present study, all patients evaluated in our ACHD outpatient clinic from September 2014 to February 2021 were screened. Patients with a patent foramen ovale, cardiomyopathies and congenital arrhythmias without any structural abnormalities were excluded. CHD distribution across patients population is depicted in Supplementary Table 1. Among the remaining ACHD patients, those who were treated by other Institutions and referred to our center just for a single consultation were also excluded.

### Baseline clinical and echocardiographic data

Clinical, imaging and operation details were prospectively entered into a digitalized medical records database dedicated to cardiovascular patients (SI-cardio, GESI, Rome, Italy). From the above-mentioned database, we obtained the report of the first clinical outpatient evaluation that included complete medical history, vital signs, electrocardiogram (ECG), complete echocardiography. According to our institutional clinical pathway dedicated to ACHD patients (8), clinical assessment, ECG reading, and echocardiograms are directly performed and reported from experienced cardiologists specialized in the imaging and care of this patients’ population (FG, AD, and RL) and all patients that require an intervention are discussed in Heart Team, as previously described (9).

Comphrensive echocardiography was performed using commercially available ultrasound systems (Toshiba Artida, Tokyo, Japan; Philips Epiq 7, Amsterdam, Netherlands) equipped with 3.5 MHz or M5S transducers as previously reported (10) and was also used to record the presence and degree of VHD.

For each enrolled patient the clinical records were revised to extract the following data:

- Clinical findings: age, gender, CHD diagnosis at birth, number of cardiac interventions performed before our first evaluation, age at cardiac intervention(s), presence of genetic syndrome, severity of the CHD lesion (assessed according to the classification proposed in the latest ESC Guidelines) (11), previous pacemaker or implantable cardioverter defibrillator (PM/ICD) implantation, O_2_ saturation at rest (%Sat O_2_), New York Heart Association (NYHA) functional class, symptoms.
- ECG findings: rhythm; PR interval (msec); presence of right or left bundle branch block; QRS interval (msec).
- Echocardiographic findings: left ventricle ejection fraction (LVEF) assessed with biplane Simpson’s method (applied also for the evaluation of the systemic right ventricular function and the systolic function of single ventricle physiology patients), right ventricle systolic function expressed by tricuspid annular plane systolic excursion (TAPSE), parameters of diastolic function (E/A; E/e’, left atrium volume index, LAVi), right ventricle systolic pressure (RVSP), degree of valvular stenosis or regurgitation as defined by a multiparametric approach according to the current best practices recommended for VHD patients (12–15).

All forms of VHD were included: primary valvular disease (bicuspid aortic valve, Ebstein’s anomaly, etc.), valvular lesions secondary to the sequelae of the intervention performed for CHD (pulmonary regurgitation from repaired Tetralogy of Fallot, valvular insufficiency from previous valvulotomy etc.) as well as functional VHD (atrioventricular valve regurgitation from systemic right ventricle, univentricular heart or Fontan repair etc.) and percutaneous/surgical prosthesis dysfunction (11).

Severe valvular heart disease (S-VHD) was defined as the presence of at least one valve with severe dysfunction according to the latest guidelines. The grading of the severity of VHD is reported in Supplementary Table 2. Multiple VHD was defined as the presence of more than one valve with severe lesion. Severe VHD distribution according to the etiology has been depicted in Supplementary Table 3.

### Clinical follow-up and study endpoints

Clinical data at follow-up were collected through the medical records of the last evaluation at our ACHD Outpatient Clinic, or at the last hospital admission or by phone contact.

The endpoint of the study was the composite of cardiac death and/or cardiac hospitalization. Cardiac death was defined as any death without clear non-cardiac cause. Cardiac hospitalization was defined as any hospitalization due to heart failure and/or arrhythmias.

The occurrence of major arrhytmias not requiring hospitalization as well as the rate of cardiac interventions (percutaneous or surgical) were also recorded.

The study conforms to the ethical guidelines of the 1975 Declaration of Helsinki and was approved by the Institutional Ethical Committee (protocol number 4742).

### Statistical analysis

Continuous variables normally distributed are presented as mean ± standard deviation whereas non-normally distributed data are presented as median and interquartile range (IQR). Categorical variables are expressed as frequencies and percentages.

The comparison of baseline characteristics between patients with S-VHD (no severe VHD: NS-VHD) was performed by the unpaired Student’s *T* test (for normally distributed continuous variables), Mann–Whitney *U* test (for non-normally distributed continuous variables) and Chi-square test or Fisher’s exact test, as appropriate (for categorical variables).

Cumulative event-free survival rates for the population, stratified by the presence of severe VHD, were calculated using the Kaplan–Meier method. The log-rank test was used to compare the two groups. Cox proportional hazards regression analysis was used to assess the association between clinical and echocardiographic parameters with the composite study endpoint. Exposure to percutaneous or surgical interventions was included in the analysis as a binary time-dependent term. The hazard ratio (HR) and 95% confidence intervals (CIs) were calculated for each variable. The proportional hazards assumption was verified based on Schoenfeld residuals. The entry criteria for the multivariate regression analysis were a significant association in univariate analysis (*p* < 0.05) and an amount of missing values that did not exceed 5% of the total study population. A minimum tolerance level of 0.5 was established to avoid multicollinearity between covariates. Moreover, to investigate the incremental prognostic value of S-VHD on the top of variables included in the multivariate analysis, the likelihood ratio test for nested models was performed. The change in global Chi-square was calculated and reported.

As secondary analysis, the occurrence of cardiovascular mortality and cardiovascular hospitalization under medical management (censoring patients at the time of interventions) was also performed, using Cox proportional hazard regression analysis.

All tests were two-sided and *p*-values < 0.05 were considered statistically significant. Statistical analysis was performed using SPSS version 25.0 (IBM Corporation, Armonk, NY, USA) and R version 4.0.1 (R Foundation for Statistical Computing, Vienna, Austria).

## Results

### Characteristics of the study population

**FIGURE 1:**
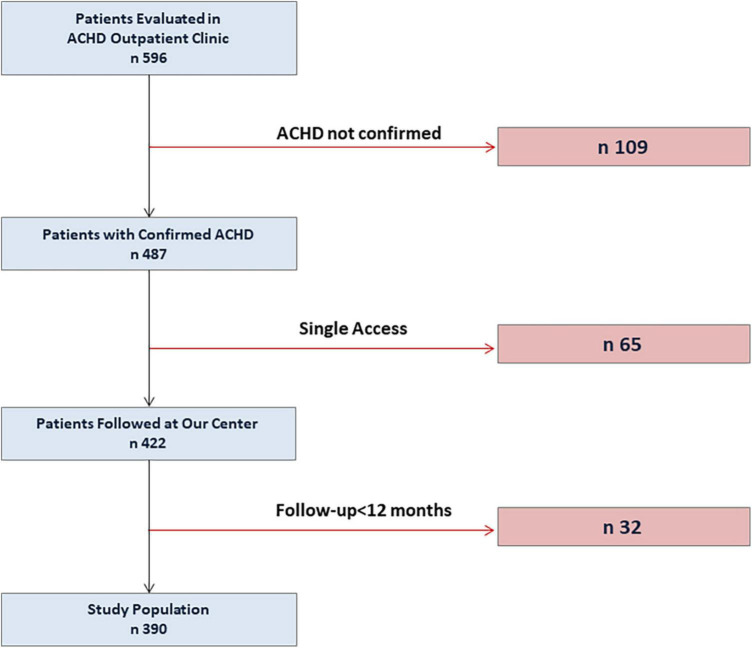
Study flow-chart. ACHD, Adult congenital heart disease.

During the study period, 596 patients were evaluated in our ACHD Outpatient Clinic. After exclusion of patients with patent foramen ovale, cardiomyopathies or congenital arrhythmias, and of those who had a single consultation, 422 ACHD patients were eligible for the study. Out of them, 32 patients had a follow-up time < 12 months and were excluded from the present study. Thus, a total of 390 patients constituted the study population. The flow chart of the study is graphically summarized in Figure 1.

The clinical characteristics of the study population are reported in Table 1. Overall, median age was 34 years (range 26–46) and 189 (49%) patients were male. Most patients (*n* = 256, 66%) underwent corrective or palliative procedure during childhood and 30 (8%) had a PM/ICD implantation. At our first evaluation, 146 (37%) patients presented with NYHA functional class > II and 22 (6%) had atrial flutter/atrial fibrillation (Af/Afib).

**TABLE 1:** Baseline characteristics of the study population.

Variables	Total (*n* = 390)
Age (years)	34 (26–46)
Male (n, %)	189 (49)
**Complexity of CHD (n, %)**	
Mild	159 (41)
Moderate	191 (49)
Severe	40 (10)
**Surgery in pediatric age (n, %)**	
None	184 (47)
Corrective surgery	179 (46)
Palliative surgery	27 (7)
Previous implantation of PM/ICD (n, %)	30 (8)
Af/Afib (n, %)	22 (6)
HR (bpm)	73 ± 13
QRS (ms)	105 (94–126)
Sat O_2_(%)	97 ± 5
NYHA class II-IV	146 (37)
LVEF (%)	61 ± 7
LAVi (ml/m2)	31 (24–43)
E/E′ ratio	7 (5–8)
S-VHD (n, %)	101 (26)
TAPSE (mm)	22 ± 5
RVSP (mmHg)	30 (25–40)
RVSP > 45 mmHg (n, %)	29 (7)

Values are expressed ad mean ± SD, median (IQR) or n (%).

Af/Afib, atrial flutter/atrial fibrillation; CHD, congenital heart disease; HR, heart rate; ICD, implantable cardioverter defibrillator; LAVi, left atrium volume index; LVEF, left ventricular ejection fraction; NYHA, New York Heart Association; PM, pacemaker; RVSP, right ventricle systolic pressure; Sat O_2_, oxygen saturation; TAPSE, tricuspid annular plane systolic excursion.

Nearby half of the population had moderate CHD (191 patients, 49%) and CHD was graded as severe in 40 patients (10%). Supplementary Figure 1 shows the distribution of severe CHD.

**FIGURE 2:**
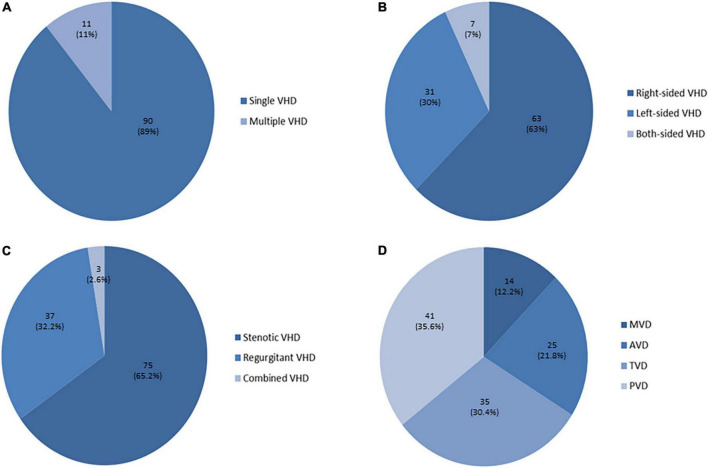
Distribution of severe heart valve lesions. **(A)** Among patients with severe valvular heart disease (S-VHD), 90 (89%) had single S-VHD (= 1 valve with severe dysfunction), while 11 (11%) had multiple S-VHD (≥ 2 valves with severe lesions). **(B)** Right-sided S-VHD (63 patients, 63%) were prevalent as compared to left-sided S-VHD (31 patients, 30%), while in few cases both-sided S-VHD were detected (7 patients, 7%). **(C)** When considering patients with multiple VHD (= more than one valve with severe dysfunction in the same patient), a total of 115 severe valvular dysfunctions were detected among the 101 patients with S-VHD. Severe regurgitant lesions were prevalent (75) as compared to severe stenotic lesions (37), while in three cases severe combined VHD was present (2 patients with severe pulmonary valve steno-insufficiency and 1 with severe mitral valve steno-insufficiency). **(D)** The pulmonary valve was the most frequently affected by severe stenosis and/or regurgitation (S-PVD, *n* = 41), followed by the tricuspid valve (S-TVD, *n* = 35). Severe aortic valve (S–AV) and mitral valve (S–MV) stenosis and/or regurgitation were less common (25 and 14 respectively).

Severe valvular heart disease (S-VHD) was present in 101 patients (25.9%). The pulmonary valve was the valve more frequently affected by severe stenosis and/or regurgitation (*n* = 42, 40.5%), while the single most common valve lesion was severe tricuspid regurgitation (*n* = 31, 30.7%). The distribution of severe valvular lesions is illustrated in Figure 2. Among patients with S-VHD, 36 underwent a surgical procedure and 6 patients a percutaneous intervention.

### Characteristics of patients with severe valvular heart disease

Table 2 shows the comparison between patents with S-VHD and those with NS-VHD. Age at first visit was significantly higher in S-VHD patients (*p* < 0.001), who also were more likely to have a moderate or severe underlying CHD (*p* < 0.001) and to have undergone corrective or palliative surgery in pediatric age (*p* = 0.014). S-VHD patients had statistically significant lower %Sat O_2_ (*p* < 0.011) and were more often in NYHA functional class > II (*p* < 0.001).

**TABLE 2:** Comparison between severe valvular heart disease (S-VHD) vs. NS-VHD patients.

Variables	S-VHD (*n* = 101)	NS-VHD (*n* = 289)	*P*-value
Age (years)	40 (30–54)	33 (24–44)	**<0.001**
Male (n, %)	49 (49)	140 (48)	0.990
**Complexity of CHD (n, %)**			
Mild	23 (23)	136 (47)	**<0.001**
Moderate	63 (62)	128 (44)	
Severe	15 (15)	25 (9)	
**Surgery in pediatric age (n, %)**			
None	37 (37)	147 (51)	**0.0014**
Corrective surgery	53 (53)	127 (44)	
Palliative surgery	11 (10)	15 (5)	
Age at first surgery	5 (1.0–13.25)	3 (0.0–10.5)	0.11
Previous implantation of PM/ICD (n, %)	10 (10)	20 (7)	0.343
Af/Afib (n, %)	15 (15)	7 (3)	**<0.001**
HR (bpm)	73 ± 15	72 ± 12	0.49
QRS (ms)	117 (101–149)	103 (93–119)	**<0.001**
Sat O_2_(%)	95 ± 6	97 ± 4	**0.011**
NYHA class II-IV (n, %)	57 (57)	89 (31)	**<0.001**
LVEF (%)	60 ± 6	62 ± 7	**0.027**
LAVi (ml/m2)	41 (27–61)	29 (23–40)	**<0.001**
E/E′ ratio	7 (5–10)	6 (5–8)	0.344
TAPSE* (mm)	20 ± 6	22 ± 5	**0.006**
RVSP (mmHg)	40 (30–55)	30 (25–35)	**<0.001**
RVSP > 45 mmHg (n, %)	17 (17)	12 (4)	**<0.001**

Values are expressed ad mean ± SD, median (IQR) or n (%).

Bold values indicate statistical significance at the *p* < 0.05 level.

*TAPSE was available in 326 patients (84% of the overall population).

Af/Afib, atrial flutter/atrial fibrillation; CHD, congenital heart disease; HR, heart rate; ICD, implantable cardioverter defibrillator; LAVi, left atrium volume index; LVEF, left ventricular ejection fraction; NS-VHD, non-severe valvular heart disease; NYHA, New York Heart Association; PM, pacemaker; RVSP, right ventricle systolic pressure; Sat O_2_, oxygen saturation; S-VHD, severe valvular heart disease; TAPSE, tricuspid annular plane systolic excursion.

At ECG, S-VHD patients presented more frequently with Af/Afib rhythm at first evaluation (*p* < 0.001) and had longer QRS duration (*p* < 0.001) as compared to NS-VHD patients.

At echocardiography, patients with S-VHD had lower LVEF (*p* = 0.027), lower TAPSE (*p* < 0.006), larger LAVi (*p* < 0.001), and increased RVSP (*p* < 0.001) as compared to NS-VHD patients.

### Impact of severe valvular heart disease on outcome

Over a median follow-up time of 26 months (IQR: 12–48), the study endpoint occurred in a total of 76 patients (19.5%). Cardiovascular death occurred in 8 patients with 2 cases of sudden cardiac death. A cardiac intervention was performed in 78 patients (median time to intervention being 10 months, range 4–29). Patients with S-VHD underwent more frequently percutaneous or surgical intervention, as compared with NS-VHD (41.6 vs. 12.5%, *p* < 0.001).

Arrhythmias occurred more often in patients with S-VHD than NS-VHD (32.7 vs. 15.2%, *p* < 0.001).

**FIGURE 3:**
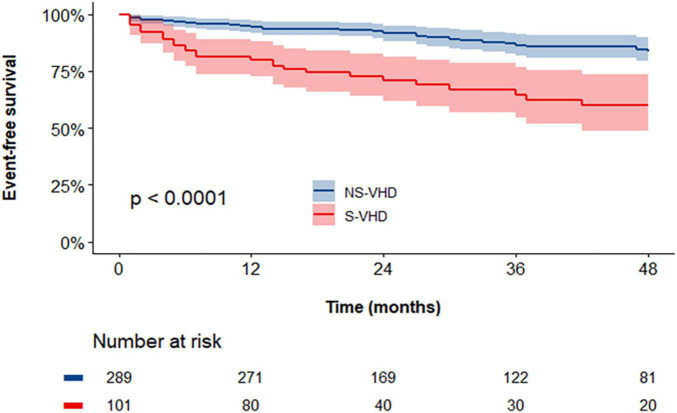
Kaplan–Meier curves assessing the cumulative event-free survival rates in severe valvular heart disease (S-VHD) and NS-VHD populations. S-VHD, severe valvular heart disease; NS-VHD, non-severe valvular heart disease.

The cumulative endpoint-free survival was significantly lower in patients with S-VHD *vs.* NS-VHD [Figure 3, 59 vs. 83% (Log rank *p* < 0.001)]. Supplementary Table 4 summarizes the prevalence of the single endpoints in the overall population as well as in S-VHD and NS-VHD groups.

At univariable Cox regression analysis (Table 3), S-VHD was significantly associated with an increased risk of the study endpoint [3.483 (2.086–5.816); *p* < 0.001]. In addition, age, severe CHD, Af/Afib at first visit, %Sat O2, NYHA class ≥ II, LVEF, TAPSE and increased RVSP (> 45 mmHg) were also associated with the study endpoint. On multivariable analysis (Table 3), the association between S-VHD and the study endpoint remained significant [HR: 1.925 (1.133–3.271); *p* = 0.015], after adjustment for age, severe CHD, Af/Afib at first visit, %Sat O_2_, NYHA class ≥ II, LVEF and increased RVSP. Notably, the likelihood ratio test demonstrated an incremental prognostic value by incorporating S-VHD in the multivariable model (changes in *X*^2^ = 5.70; *p* = 0.017) (Supplementary Figure 2).

**TABLE 3:** Univariable and multivariable Cox regression analysis.

	Univariable analysis		Multivariable analysis	
Variables	OR (95% CI)	*P*-value	OR (95% CI)	*P*-value
Age (years)	1.037 (1.020–1.054)	**<0.001**	1.022 (1.002–1.041)	**0.029**
Male	0.981 (0.623–1.546)	0.935		
Severe CHD	3.483 (2.086–5.816)	**<0.001**	1.332 (0.663–2.673)	0.421
Surgery in pediatric age	1.376 (0.865–2.186)	0.177		
Af/Afib	6.444 (3.224–12.877)	**<0.001**	3.214 (1.408–7.333)	**0.006**
HR (bpm)	1.011 (0.994–1.029)	0.209		
QRS duration (ms)	1.007 (0.999–1.014)	0.075		
Sat O_2_ (%)	0.934 (0.914–0.955)	**<0.001**	0.956 (0.926–0.986)	**0.005**
NYHA class II–IV	4.224 (2.613–6.827)	**<0.001**	2.246 (1.270–3.974)	**0.005**
Surgical or percutaneous intervention*(time-dependent)*	1.503 (0.745–3.031)	0.255		
LVEF (%)	0.922 (0.896–0.948)	**<0.001**	0.952 (0.918–0.987)	**0.008**
LAVi (ml/m2)	1.000 (0.998–1.003)	0.657		
E/E′ ratio	1.049 (0.932–1.181)	0.426		
S-VHD	3.258 (2.073–5.121)	**<0.001**	1.925 (1.133–3.271)	**0.015**
TAPSE (mm)	0.901 (0.856–0.949)	**<0.001**		
RVSP > 45 mmHg	1.029 (1.017–1.040)	**<0.001**	2.023 (0.955–4.286)	0.066

Bold values indicate statistical significance at the *p* < 0.05 level.

Af/Afib, atrial flutter/atrial fibrillation; CHD, congenital heart disease; HR, heart rate; LAVi, left atrium volume index; LVEF, left ventricular ejection fraction; NYHA, New York Heart Association; Sat O_2_, oxygen saturation; S-VHD, severe valvular heart disease; TAPSE, tricuspid annular plane systolic excursion; RVSP, right ventricle systolic pressure.

The association of each subgroup of S-VHD (defined according to the affected valve and the type of valvular lesion), with outcomes is reported in Supplementary Figure 3. Each type of S-VHD was individually associated with the endpoint. After adjustment for significant clinical and echocardiographic variables (age, severe CHD, Af/Afib, NYHA ≥ II, Sat O_2_, LVEF and RVSP > 45 mmHg), isolated S-VHD (*p* = 0.049), left-sided VHD (*p* = 0.007), and regurgitant S-VHD (*p* = 0.016) retained their independent association with outcomes.

There was no significant difference in adverse outcomes occurrence between S-VHD patients who received corrective intervention during follow-up and those who did not [14 (33%) vs. 23 (39%), respectively, *p* = 0.56]. However, in the subgroup of the S-VHD population that underwent surgical/percutaneous procedure, most of the events [10, (71%)] occurred before the intervention.

To further confirm the prognostic value of S-VHD on the clinical evolution, the occurrence of cardiac mortality or cardiac hospitalization without cardiac operations was examined. After a median follow-up of 23 months (range: 9–44), 68 events occurred. At univariable Cox regression analysis, S-VHD was significantly associated with outcomes in patients medically treated [HR: 3.473 (2.144–5.627); *p* < 0.001]. The adjustment for significant clinical and echocardiographic variables (age, severe CHD, Af/Afib, NYHA class ≥ II, LVEF and increased RVSP) did not affect the powerful association of S-VHD with the occurrence of the composite endpoint under medical management [HR: 1.920 (1.093–3.373); *p* = 0.023].

**FIGURE 4:**
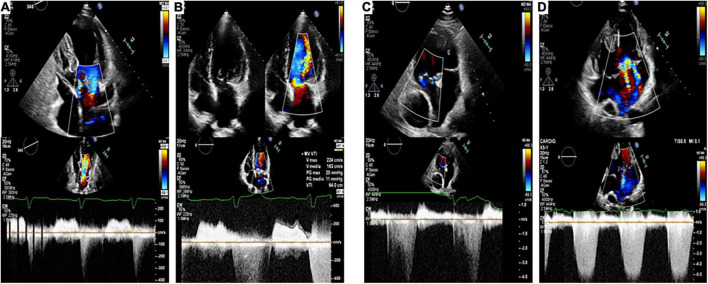
Echocardiographic images (Top: apical 4-chamber views, Bottom: CW Doppler through mitral valve and systemic AV valve) of four patients included in our study, coupled according to the ACHD [**(A,B)**: repaired partial atrioventricular septal defect; **(C,D)**: Fontan circulation], with (Panel **B,D**) and without (Panel **A,C**) associated S-VHD. This is illustrative of our findings, since patients **(B–D)** experienced hospitalization for heart failure. while patients **(A–C)** had an unremarkable clinical course.

The Figure 4 is an illustrative example of four patients included in our study, coupled according to the ACHD.

## Discussion

The major and novel finding of our study in a contemporary real-world ACHD population is that the presence of severe VHD impacts prognosis, being independently associated with the risk of cardiac mortality and hospitalization. This highlights the need for careful VHD characterization in ACHD outpatient clinics and call for improvement in their management.

The management of patients with ACHD represents an expanding clinical field, with constantly increasing numbers, mainly due to the success of cardiac surgery and interventions in children (16).

These patients, however, cannot be considered cured, as most of them will suffer the long-term sequelae of their birth defect and/or the surgical interventions performed in childhood, which can take decades to manifest (17). The clinical complexity of the ACHD population, indeed, is increasing over time (1, 4, 18), with HF and arrhythmias representing the major causes of death or re-admission for these patients during adult life (18–20) and VHD are commonly found and frequently require an intervention (21, 22). To date, however, most data come from registries or surveys (based on large administrative databases, general electronic health records and death certificates) that cannot provide detailed information about important clinical features regarding baseline disease severity characterization and outcome measures. More importantly, these are frequently descriptive analyses in which factors often associated to ACHD, as the presence of VHD, are not evaluated as covariates. All these limits make the real burden of VHD on ACHD difficult to ascertain.

In this context, the major strength of our study is that we carefully collected clinical, ECG and echocardiographic data in a contemporary real-world population of ACHD patients followed at our dedicated outpatient clinic, with the specific aim to assess the role of severe VHD on prognosis. We found that half of the population had a VHD at least moderate at the first evaluation in our center. This is of particular importance, as moderate valve dysfunction has been shown to be detrimental in other clinical settings (23) as well as in ACHD (24). Moreover, the median age of our population at first visit was 34 years, thus the chance of progression of the VHD is expected to be high. As much as a quarter of patients presented at their first evaluation in our dedicated adult outpatient clinic with a severe valve dysfunction, that is expected to determine a major impact on the overall CHD hemodynamics. These patients were older, presented with more advanced NYHA functional class and more often had an underlying moderate or severe CHD, in line with recent studies reporting the increasing complexity of ACHD patients (1, 4–17). Moreover, patients with severe VHD were more prone to develop arrhythmias and more often required interventions/reinterventions, confirming previous observations (25–27).

The major finding of our study is that severe VHD at presentation was independently associated with an adverse outcome during the clinical course. S-VHD adds strong prognostic value to the multivariate model including significant clinical and echocardiographic variables. In particular, S-VHD had an independent prognostic impact on outcome while severe CHD did not. This is worth noting, since most data suggest that cardiovascular death and complications are associated with the severity of underlying CHD (21, 28, 29). A study (21) evaluating the trends in hospitalizations for ACHD (2003–2012), stratified into simple and complex disorder, showed that HF, respiratory disorders, and arrhythmias were the top three reasons for admission among patients with complex ACHD while VHD was among the three top causes for admission among patients with simple ACHD without atrial septal defect/patent foramen ovale.

However, these studies did not stratify patients for the degree of VHD while our study showed the relevance of the severity of the valvular heart lesion on ACHD outcome. This data is of particular importance as most studies reporting on VHD in CHD patients comes from national registries and surveys with the valve dysfunction often reported as a dichotomous variable and no mention on the type and degree of valvular lesion, or the affected valve or the underlying etiology. Of note, our study reveals the striking difference between the typical scenario of adult cardiology, in which VHD poses a significant burden dominated by far by aortic stenosis and mitral regurgitation (30) and ACHD valvular abnormalities. In fact, ACHD comprise a wide spectrum, with extremely heterogeneous anatomy, physiology and surgical history, including systemic right ventricles, systemic single ventricle and Fontan palliation. Due to this complexity, ACHD-related VHD are difficult to categorize as primary, post-surgical or functional lesions. Indeed, on an individual basis, patients often fall in more than one category. Thus, whether there is a different prognostic role for primitive vs. post-surgical or functional VHD cannot be deduced from our study.

In this context, right-sided valvular lesions were prevalent in our study but left-sided VHD (including all systemic atrio-ventricular valves) retained their independent association with outcomes. These findings highlight the importance of considering valvular heart lesions in ACHD according to the functional, rather than the anatomical classification.

### Limitations

Some limitations of the present study should be acknowledged. Firstly, we included a wide spectrum of congenital heart diseases patients with heterogeneous valvular lesions. However, this is a problem commonly encountered in studies on ACHD, considering that most CHD are rare diseases. In addition, this is an observational study conducted in a tertiary referral center for ACHD and VHD, thus our population could not be representative of average ACHD patients. Moreover, we did not systematically collect cardiac magnetic resonance (CMR) data that is the gold standard for the assessment of most ACHD patients. However, the presence and degree of VHD was carefully established according to the most recent echocardiography guidelines and echo studies were personally performed by experienced team in the imaging and care of VHD and ACHD. Moreover we did perform CMR in every patient with pulmonary regurgitation to quantify its severity by means of regurgitant fraction. It is also worth noting that echocardiography is still the gold standard for the assessment of valvular stenosis.

## Conclusion

Our study shows that the occurrence of S-VHD in ACHD patients is a major threat, being a significant independent predictor of hospitalization or death. The prognostic value of severe valve dysfunction is incremental above other established prognostic markers and consistent independently from CHD severity.

## Data availability statement

The raw data supporting the conclusions of this article will be made available by the authors, without undue reservation.

## Ethics statement

The studies involving human participants were reviewed and approved by Ethic Committee Fondazione Policlinico Universitario Agostino Gemelli, n°4742. Written informed consent for participation was not required for this study in accordance with the national legislation and the institutional requirements.

## Author contributions

FG, GI, MCM, and RL: study design, data collection, and statistical analysis. All authors contributed to the drafting and revision of the manuscript, to data analysis improvement and discussion development.
