# Supplementary material for: Impact of severe valvular heart disease in adult congenital heart disease patients
Source: Front Cardiovasc Med. 2022 Nov 29;9:983308. doi: 10.3389/fcvm.2022.983308 (PMC9744774; doi:10.3389/fcvm.2022.983308)
Supplement: Supplementary file 1 [file Data_Sheet_1.docx]

**SUPPLEMENTARY MATERIALS**

**Table S1. Congenital heart disease distribution.**

| Congenital Heart Disease | n (%)  tot 390 |
| --- | --- |
| Atrial septal defect | 44 (11.3) |
| Atrial septal defect + APVR | 14 (3.6) |
| Ventricular septal defect | 41 (10.5) |
| Bicuspid aortic valve | 42 (10.8) |
| Unicuspid aortic valve | 2 (0.5) |
| Tetralogy of Fallot | 44 (11.3) |
| Unrepaired Tetralogy of Fallot | 2 (0.5) |
| Trilogy of Fallot | 6 (1.5) |
| Great arteries transposition | 27 (6.9) |
| Fontan palliation | 20 (5.1) |
| Univentricular heart | 2 (0.5) |
| Hetrotaxy syndrome | 5 (1.3) |
| Glenn shunt | 8 (2) |
| Pulmonary Atresia | 10 (2.6) |
| Truncus arteriosus | 3 (0.8) |
| Eisenmerger syndrome | 10 (2.6) |
| Double outlet right ventricle | 10 (2.6) |
| Atrioventricular canal defect | 28 (7.2) |
| Ebstein anomaly | 14 (3.6) |
| Pulmonary stenosis | 15 (3.8) |
| Aortic arch anomalies | 3 (0.8) |
| Subaortic stenosis | 7 (1.8) |
| Pervious ductus arteriosus | 8 (2) |
| Aortic coartation | 9 (2.3) |
| Anomalous origin of coronary arteries | 3 (0.8) |
| Isolated APVR/persistent LSVC | 7 (1.8) |
| Aortic-pulmonary window | 2 (0.5) |
| Dysplastic tricuspid valve | 4 (1) |

*Legend:* Distribution of congenital heart disease across the study population. *In case of coexistent multiple congenital heart defects, patients were categorized according to the most severe disease (either for anatomy or hemodynamic impact).*

*Abbreviations:* APVR: anomalous pulmonary vein return; LSVC: left superior vena cava.

**Table S2. Definition of Severe VHD.**

|  | Severe Native Valve Regurgitation | Severe Native Valve  Stenosis | Severe Prosthetic Valve Regurgitation | Severe Prosthetic Valve Obstruction |
| --- | --- | --- | --- | --- |
| Mitral Valve | -VCW ≥ 7 mm  -EROA ≥ 0.40 cm^2^  -Systolic flow reversal in pulmonary veins  -E wave > 1.2 m/s  -RVol ≥ 60 mL  -RF ≥ 50% | -MVA < 1 cm^2^  -Mean gradient > 10 mmHg  -PAP > 50 mmHg | -Peak velocity ≥ 1.9 ms/s  -Mean gradient ≥ 5 mmHg  -VCW > 6 mm  -Systolic flow reversal in pulmonary veins  -Circumferencial extent of paravalvular  regurgitation ≥ 30%  -EROA ≥ 0.40 cm^2^  -RVol ≥ 60 mL  -RF ≥ 50% | -Peak velocity ≥ 2.5 m/s  -Mean gradient ≥ 10 mmHg  -EOA < 1 cm^2^  -Reference EOA-measured EOA >0.35  -Measured EOA < reference EOA - 2SD  -DVI > 2.5 |
| Aortic Valve | -VCW > 6 mm  -EROA ≥ 0.30 cm^2^  -Holodiastolic flow reversal in descending aorta  -RVol ≥ 60 mL  -RF ≥ 50% | -Peak velocity ≥ 4.0 m/s  -Mean gradient ≥ 40 mmHg  -AVA < 1.0 cm^2^  -Indexed AVA  < 0.6 cm^2^  -Velocity ratio <0.25 | -PHT < 200 ms  -VCW > 6 mm  -Holodiastolic flow reversal in descending aorta  -Circumferencial extent of paravalvular  regurgitation ≥ 30%  -EROA ≥ 0.30 cm^2^  -RVol ≥ 60 mL  -RF > 50% | -Peak velocity ≥ 4.0 m/s  -Mean gradient ≥ 35 mmHg  -EOA < 0.8 cm^2^  -Reference EOA-measured EOA >0.35  -Measured EOA < reference EOA - 2SD  -Velocity ratio <0.25 |
| Tricuspid Valve | -VCW ≥ 7 mm  -PISA radius > 0.9  -EROA ≥ 0.40 cm^2^  -Systolic flow reversal in hepatic veins  -E wave > 1 m/s  -RVol ≥ 45 mL | -Mean gradient ≥ 5 mmHg  -Inflow VTI > 60 cm  -PHT ≥ 190 ms  -TVA by continuity equation ≤ 1 cm^2^ | -VCW ≥ 7 mm  -Elevated pressure gradient  -Systolic flow reversal in hepatic veins | -PHT ≥ 130 ms  -DVI ≥ 2  -Peak velocity ≥ 1.9 m/s  -Mean gradient ≥ 6 mmHg |
| Pulmonary Valve | -PR jet width/pulmonary  annulus > 0.7  -PHT < 100 ms  -RF > 40% | -Peak velocity 4 m/s  -Peak gradient > 64 mmHg | -Diastolic flow reversal in pulmonary artery  -PHT < 100 ms | -PHT ≥ 230 ms  -Peak velocity:  Bioprosthesis ≥ 3.2 m/s  Homograft ≥ 2.5 m/s  -Mean gradient:  Bioprosthesis ≥ 20 mmHg  Homograft ≥ 15 mmHg |

*Legend:* The grading of the VHD was multiparametric as suggested by the latest Guidelines and included the repercussion on chamber size and pulmonary pressure. Here are reported the main echocardiographic parameters considered for the definition of severe VHD.

*Abbreviations:* AVA: aortic valve area; DVI: Doppler velocity index; EOA: effective orifice area; EROA: effective regurgitant orifice area; MVA: mitral valve area; PAP: pulmonary artery pressure; PHT: pressure half time; PISA: Proximal Isovelocity Surface Area; PR: pulmonary regurgitation; RF regurgitant fraction; RVol: regurgitant volume; SD: standard deviation; TVA: tricuspid valve area; VCW: vena contracta width; VHD: valvular heart disease.

**Table S3. Main underlying mechanisms determining S-VHD in the study population.**

| S-VHD etiology | n (%)  tot 101 |
| --- | --- |
| Primary VHD | 45 (44.5%) |
| Secondary VHD – surgical sequelae | 38 (37.6%) |
| Secondary VHD – prosthesis dysfunction | 2 (2%) |
| Secondary VHD – functional | 16 (15.9%) |

*Legend:* Distribution of valvular heart disease main etiologies in patients with severe valvulopathy.

*Abbreviation:* S-VHD: severe valvular heart disease.

**Table S4. Total number of events in the overall population and two sub-groups.**

| Outcome | Total population  (n=390) | S-VHD  (n=101) | NS-VHD  (n=289) | *p-value* |
| --- | --- | --- | --- | --- |
| Cardiac hospitalization (n,%) | 74 (19.0) | 36 (35.6) | 38 (13.1) | <0.001 |
| Cardiac death (n,%) | 8 (2.1) | 6 (6.1) | 2 (0.7) | 0.001 |

*Legend:* Distribution of the singular elements of the composite primary endpoint according to the presence or not of S-VHD.

*Abbreviations:* NS-VHD: NS-VHD: non-severe valvular heart disease; S-VHD: S-VHD: severe valvular heart disease.

**Figure S1. Distribution of severe ACHD.**

*Legend:* Prevalence of severe adult congenital heart disease (ACHD) types defined according to 2020 ESC Guidelines for the management of ACHD.

*Abbreviations:* ccTGA: congenitally corrected transposition of the great arteries; DORV: double outlet right ventricle; TGA: transposition of the great arteries; TOF: tetralogy of Fallot.

**Figure S2. Incremental prognostic value of S-VHD for the primary end-point.**

**
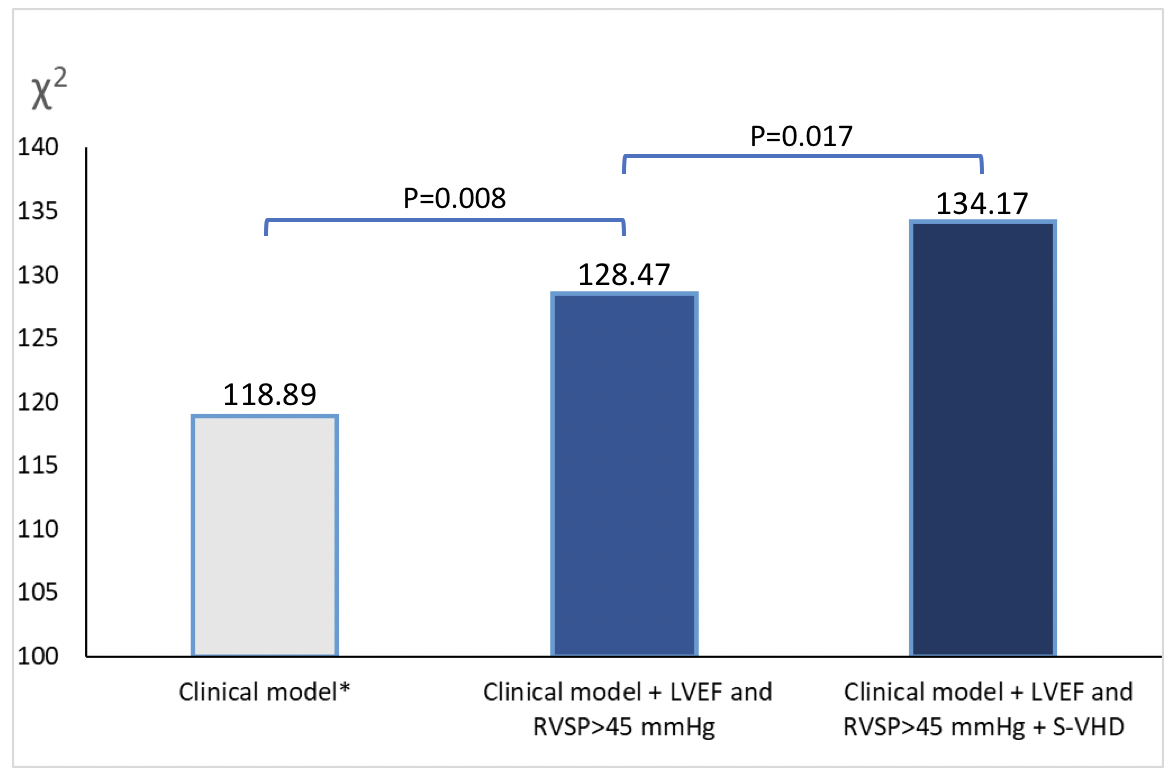
**

*Legend:* The likelihood ratio test demonstrates that the S-VHD added significant prognostic value to the multivariate model including significant clinical variables, LVEF and RVSP>45 mmHg.
* Clinical model: Age, severe CHD, Af/Afib, Sat O_2_ (%), NYHA class ≥II.

*Abbreviation:* Af/Afib: atrial fibrillation/atrial flutter*;* CHD: congenital heart disease*;* LVEF: left ventricular ejection fraction; NYHA: New York Heart Association; RVSP: right ventricular systolic pressure; Sat O_2:_ oxygen saturation; S-VHD: valvular heart disease.

**Figure S3. Cox regression analyses evaluating the association of each type of S-VHD with the primary endpoint.**

**
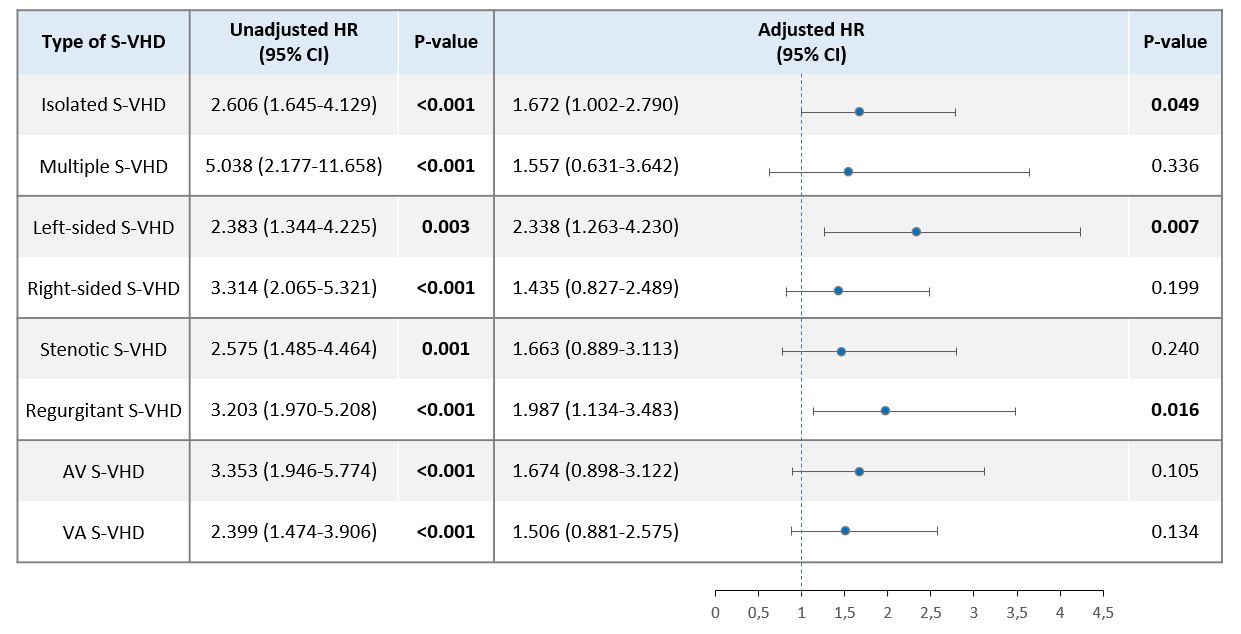
**

*Legend:* Unadjusted and adjusted HR are presented for each type of S-VHD. The adjustment was made controlling for age, severe CHD, Af/Afib, NYHA ≥II, Sat O_2_ (%), LVEF and RVSP>45mmHg. Each type of S-VHD was individually associated with the primary endpoint. After adjustment for significant clinical and echocardiographic variables, isolated S-VHD, left-sided VHD and regurgitant S-VHD retained their independent association with outcomes.

Among left-sided S-VHD we included all systemic AV valves (univentricular heart and Fontan circulation, atrial switch TGA and ccTGA)

*Abbreviations:* Af/Afib: atrial flutter/atrial fibrillation; AV: atrio-ventricular valve; CHD: congenital heart disease; HR: hazard ratio; LVEF: left ventricular ejection fraction; NYHA: New York Heart Association; Sat O_2:_ oxygen saturation; S-VHD: severe valve heart disease; VA: ventricular-arterial valve.
